# Radioactive nanosized colloids and indocyanine green identify the same sentinel lymph nodes in oral squamous cell carcinoma

**DOI:** 10.1007/s00432-023-05427-1

**Published:** 2023-10-06

**Authors:** Lukas Hingsammer, Daphne Schönegg, Thomas Gander, Martin Lanzer

**Affiliations:** 1 Medical University of Vienna, Spitalgasse 23, 1090 Vienna, Austria; 2 Department of Cranio-Maxillo-Facial and Oral Surgery, University Hospital of Zurich, Rämistrasse 100, 8091 Zurich, Switzerland

**Keywords:** Sentinel lymph-node biopsy, Indocyanine green, 99m technetium, Technetium nanocolloid, Nanosized colloids, Oral squamous cell carcinoma

## Abstract

**Purpose:**

Near-infrared fluorescence imaging using indocyanine green (ICG) combined with radioactive markers has the potential to improve sentinel lymph-node (SLN) mapping in oral squamous cell carcinoma (OSCC). This study aimed to evaluate the ability of ^99m^Tc and ICG in identifying the sentinel lymph nodes in patients with early stage OSCC.

**Methods:**

Data were collected prospectively, and a retrospective analysis of 15 patients with early stage OSCC and a cN0 neck was performed. All patients received peritumoral injection of ^99m^Tc the day before surgery and ICG was administered intraoperatively. Intentionally, the application of the two different tracers were done by two different physicians with varying degrees of experience. The number of identified lymph nodes positive for ^99m^Tc and ICG, the overlap or possible discrepancies of both methods, and the time until fluorescence signals of ICG were detected were noted.

**Results:**

In all patients, a 100% agreement in sentinel lymph-node identification was achieved, regardless of both the exact location of the peritumoral injection and the experience of the injecting surgeon. Time until ICG accumulation in the sentinel lymph node was consistently found to be between 1 and 3 min.

**Conclusion:**

ICG constitutes a viable and useful addition to ^99m^Tc for intraoperative sentinel lymph-node detection in this study.

## Introduction

The presence of metastatic cervical lymph nodes in oral squamous cell carcinoma (OSCC) is a major prognostic factor and is reported to shorten the 5-year survival by up to the half. Therefore, correct staging and detection of pathological lymph nodes is crucial (Mamelle et al. 1994; Capote et al. 2007). Clinical examination and currently available imaging methods are uncapable to detect occult neck metastases as pathological lymph nodes are found in elective neck dissection (END) specimens in 20–30% of patients with clinically unsuspicious necks (Nieuwenhuis et al. 2005; Matsubara et al. 2012; Schilling et al. 2015). However, END constitutes an overtreatment in the majority of patients. To avoid overtreatment, sentinel lymph-node biopsy (SLNB) was introduced in patients with OSCC. The first draining lymph nodes of the primary tumor are called sentinel lymph nodes (SLN). They are showing the fastest enhancement of a peritumorally injected marker substance (radioactive or fluorescent), are surgically removed and sent for histopathological examination (Alex 2004; Hingsammer et al. 2019). Histopathological examination follows a specific protocol, and is a much more thorough examination (Stoeckli 2007; Joosten et al. 2017).

In the last decade, the benefits of SLNB compared to END have been highlighted and SLNB has become the preferred procedure in the treatment of early stage oral squamous cell carcinoma (OSCC) (Broglie et al. 2013; Flach et al. 2014; Schilling et al. 2015; Hingsammer et al. 2019). Radioactive markers such as ^99m^technetium-labeled nanosized colloids (^99m^Tc) are most frequently used for sentinel lymph-node biopsy protocols in oral squamous cell carcinoma (Van Der Vorst et al. 2013; Joosten et al. 2017). Preoperatively ^99m^Tc is administered around the primary and a single-photon emission computed tomography and standard computed tomography (SPECT/CT) enables the identification of the SLN prior to surgery. Intraoperatively, the according, radioactive lymph nodes are identified by a gamma probe (Shellenberger 2006).

More recently, fluorescence-based markers like indocyanine green (ICG) have been introduced (Bredell 2010; Van Der Vorst et al. 2013; Murase et al. 2015; Süslü et al. 2022). They can be visualized intraoperatively with a near-infrared light (NIR) camera system, show rapid flare and accumulation in the sentinel lymph nodes within minutes after injection, and have been shown to be safe to use (Zhang et al. 2016). To combine the advantages of radioactive and fluorescent markers, hybrid markers (containing both ^99m^Tc and ICG) or their sequential use have been successfully used (Van Den Berg et al. 2012a; Digonnet et al. 2018). It is reported that the combined use of radiotracer and fluorescence imaging in SLNB procedures provides additional value in disease removal (Süslü et al. 2022). However, literature lacks a comparison of the two different tracers in terms of their conformity when it comes to intraoperative lymph-node identification. Up to date, it has neither been investigated if radioactive and fluorescent markers affect the same lymph nodes nor if the education level of the tracer administrating physician influences lymph-node detection.

In this study, we report on the sequential injection of radioactive and fluorescence-based SLNB markers, intending to evaluate and compare the two methods qualitatively and quantitatively regarding their ability to identify the sentinel lymph nodes in patients with early stage OSCC.

## Materials and methods

Patients aged 18 years and older scheduled for sentinel lymph-node biopsy between December 2018 and March 2022 as part of treatment for early stage oral squamous cell carcinoma (cT1-2, cN0, cM0) were enrolled in this study. All patients underwent head and neck computed tomography (CT) scan or magnetic resonance imaging (MRI) prior to surgery. Synchronous malignancy and previous surgery to the peritumoral region or the neck led to exclusion from this study.

**Fig. 1:**
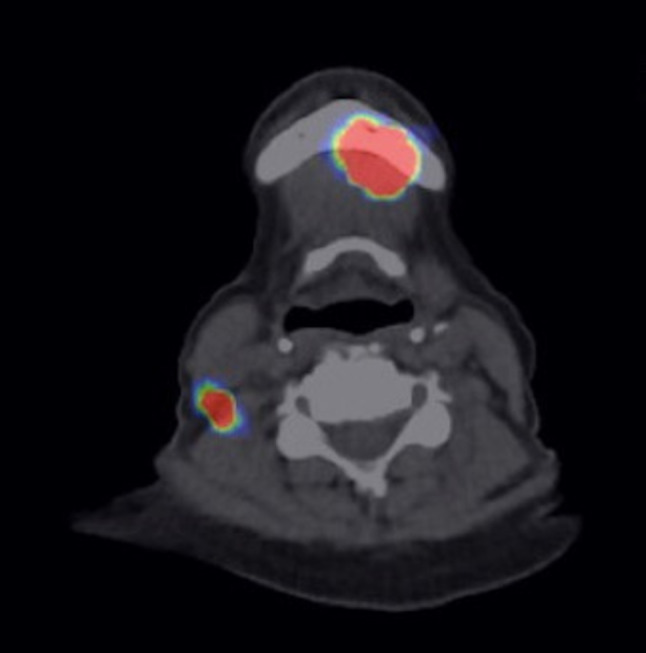
Visability of the primary and the SLN in the preoperative SPECT/CT after injection of nanosized 99mTc colloid

On the day before surgery, 80 MBq of nanosized ^99m^Tc colloid was injected in the peritumoral region by a resident (junior physician). Imaging was performed by single-photon emission computed tomography and standard computed tomography (SPECT/CT). All patients were treated under general anesthesia with standard nasal intubation. The surgical incision was planned to best expose the SLN identified on SPECT/CT (Fig. 1).

After careful preparation of a sub-platysmal flap and identification of the previously defined SLN by a gamma probe, ICG was injected around the tumor by a consultant (senior physician). A solution of ICG was made up by diluting 50 mg ICG powder with 5 ml aqueous water. The resection margins were superficially marked with diathermy as mucosal color changes after the ICG injection complicate margin identification. One milliliter of ICG solution was injected with at least five injection points along the circumference of the tumor (Bredell 2010).

**Fig. 2:**
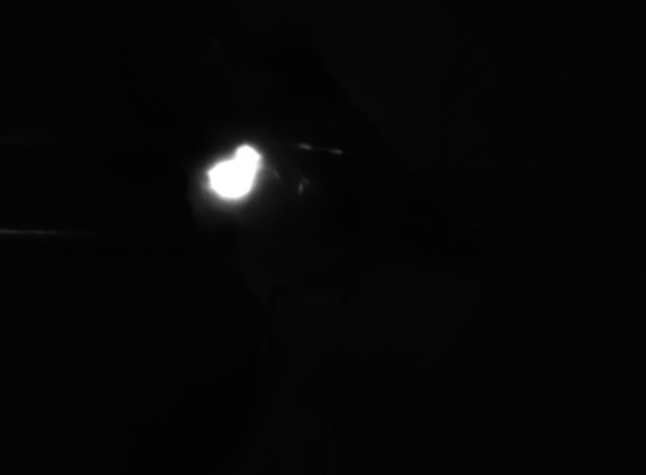
Visualization of an SLN using the NIR camera system

Fluorescence was captured by a camera system (PDE-photo-dynamic Eye, Hamamatsu Photonics Deutschland GmbH) that allows real-time NIR fluorescence imaging (Fig. 2).

To optimize the visualization of fluorescent SLN, the operating light was extensively dimmed. Time to nodal accumulation of ICG and the number of lymph nodes positive for ICG and ^99m^Tc were noted. Excised SLNs were histopathologically evaluated according to a standardized protocol using cytokeratin for immunohistochemistry (Stoeckli 2007; Hingsammer et al. 2019).

Data on tumor stages were extracted from the patients’ medical records. Exclusion criteria for administration of ICG were severe kidney failure, coronaropathy, and allergy to ICG or iodine. Statistical analysis was performed with SPSS (version 26; IBM Corp., Armonk, NY, USA) and included descriptive statistics and Mann–Whitney *U* test or Fisher’s exact test. A *p* value of < 0.05 was considered statistically significant. All patients have signed an informed consent form. The relevant ethics committee approved the conduct of this study (KEK Zürich, Approval No. 2019-01462), and this study fulfills the criteria of the Declaration of Helsinki.

## Results

15 patients, 9 female and 6 male, with a mean age of 65 years (SD: 11.2 years) were enrolled in the study. Clinical tumor stages were cT1 or cT2. All patients had a clinically unsuspicious neck (cN0) and no evidence of distant metastatic disease (cM0). Lateral tongue cancer was the most common with 11 patients affected. The remaining locations of the primary tumors are listed in Table 1.

**Table 1:** Distribution of tumor localization

Localization of primary tumor	Number of patients (percentage)
Lateral tongue	11 (73.3%)
Lower lip	1 (6.6%)
Buccal mucosa	1 (6.6%)
Floor of the mouth	1 (6.6%)
Attached gingiva of the lower jaw	1 (6.6%)

**Fig. 3:**
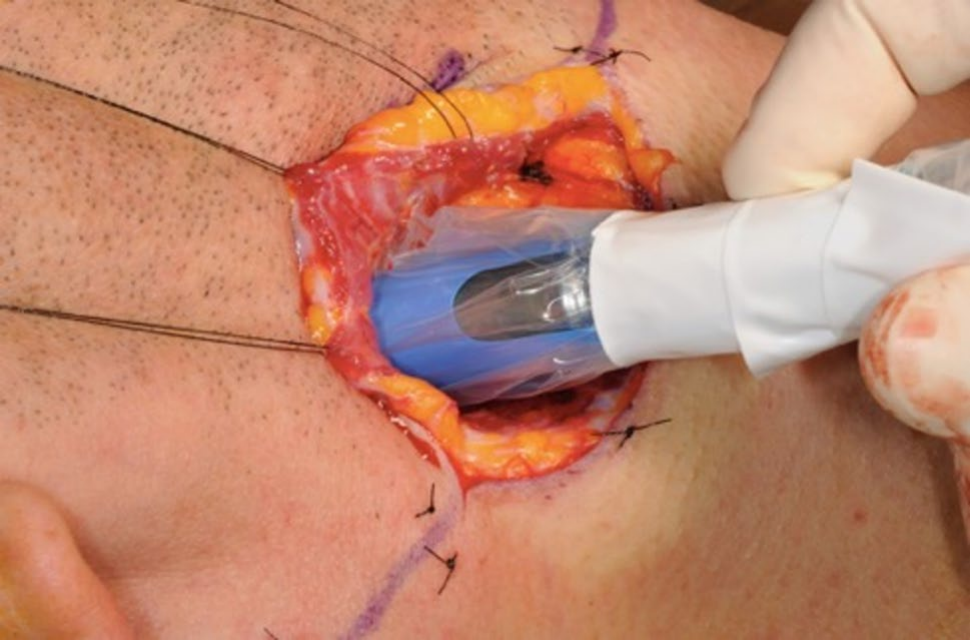
Confirmation of detected SLNs using the gamma probe

Preoperative SLN detection via SPECT/CT was successful in all cases. Following the fluorescence, dissection revealed SLNs in all patients that were confirmed by gamma probe (Fig. 3).

The number of sentinel lymph nodes varied from 1 to 4 with an average of 2. In 8 patients 1 (53%), in 2 patients 2, in 4 patients 3 and in 1 patient 4 SLNs were excised. In total, 28 SLNs were detected. SLNs were found in 4 different cervical levels (Table 2).

**Table 2:** Localization of excised sentinel lymph nodes

Cervical lymph node level	Number of lymph nodes (percentage)
Ib	3 (10.7%)
IIa	12 (42.9%)
III	12 (42.9%)
IV	1 (3.6%)

In all patients, SLN were located ipsilateral to tumor site. In 2 of 15 patients, SLNs revealed also contralateral lymphatic drainage, and thus, SLN were excised bilateral. In one of those two patients, the primary was located at the floor of the mouth and the SLNs bilateral in level III. The other patient suffered from a lateral tongue cancer and SLNs were located bilateral in level IIa. No patient showed only contralateral drainage pattern. None of the 28 resected lymph nodes had occult metastasis (0%). Table 3 gives an overview of patients and relevant parameters.

**Table 3:** Overview of patients and relevant parameters

Patient	Age	Primary	Tumor site in relation to midline	No. of detected SLN	SLN localization	Bilateral	unilateral
1	90	Lateral tongue right	Right	4	IIa right (3) III right (1)		x
2	49	Lower lip left	Left	2	Ib links (2)		x
3	71	Buccal mucosa left	Left	1	Ib links (1)		x
4	73	Lateral tongue right	Right	1	III right (1)		x
5	60	Attached gingiva of the lower jaw right	Right	1	III right (1)		x
6	46	Lateral tongue left	Left	3	IIa left (1) III left (2)		x
7	73	Lateral tongue right	Right	3	IIa right (1) III right (2)		x
8	70	Floor of the mouth	Midline	2	III right (1) III left (1)	x	
9	66	Lateral tongue left	Left	1	IIa left (1)		x
10	68	Lateral tongue right	Right	3	IIa right (2) III right (1)		x
11	54	Lateral tongue right	Right	1	III right (1)		x
12	60	Lateral tongue right	Right	1	IV right (1)		x
13	73	Lateral tongue left	Left	1	III left (1)		x
14	66	Lateral tongue right	Right	1	IIa right (1)		x
15	56	Lateral tongue left	Left	3	IIa left (2) IIa right (1)	x	

SLN mapping using ^99m^Tc registered by a gamma probe and ICG visualized by an NIR camera system marked exactly the same sentinel lymph nodes in all 15 patients, regardless of the type of marker used, the exact peritumoral injection sites, and the experience of the injecting surgeon (*p* < 0.05). The presence of ^99m^Tc and ICG in each respective sentinel lymph node was confirmed by NIR and gamma probe.

Measured by the time between peritumoral injection and first visibility of ICG by the NIR camera system, the mean time of accumulation was 1.75 min (range, 1–3 min). Regarding the fluorescence time, SLNB was completed sufficiently in all patients.

## Discussion

The benefits of SLNB compared to alternative treatment options are highlighted in numerous studies (Broglie et al. 2011; Flach et al. 2014; Schilling et al. 2015; Hingsammer et al. 2019). SLNB has been reported to have excellent detection rates of occult neck metastasis in patients with early oral cavity cancer, a sensitivity around 93%, and a negative predictive value up to 100% (Broglie et al. 2011). A major advantage of SLNB is the potential to avoid neck dissection in 60–80% of patients with early stage OSCC and unsuspicious necks (cN0) (Flach et al. 2014; Schilling et al. 2015; Hingsammer et al. 2019). Various methods and procedures have been described to be used for SLN identification (Bilde et al. 2008; Kim et al. 2021).

Although the use of radiotracers alone constitutes the most frequently applied method, the combination of ^99m^Tc- and ICG-based markers has been reported as the preferable method for SLN detection in oral cancer patients (Murase et al. 2015; Digonnet et al. 2018). Our study supports this opinion by demonstrating a 100% concordance in SLN identification with the preoperative injection of ^99m^Tc and the intraoperative injection of ICG in patients with early stage OSCC.

Our findings are particularly relevant as radio-detection and subsequent optical screening identified the same SLN in all our patients, regardless of the exact peritumoral injection site, the experience of the injecting surgeon, and regardless of time of injection. This highlights the robustness and reproducibility of the SLNB technique.

Preoperative application of radioactive tracers, such as ^9m^Tc nanocolloids, followed by lymphoscintigraphy and intraoperative usage of a gamma probe is the most frequently used method to identify sentinel nodes in patients with OSCC (van der Linden et al. 2016). A unique feature of radioactive tracers is that they can be localized by preoperative imaging and detected with the gamma camera through intact skin. Additionally, preoperative lymphoscintigraphy with intraoperative gamma-probe SLN localization can help detect atypical lymph drainage patterns (Loree et al. 2019; Kim et al. 2021).

Given the disadvantages of the standard radioactive SLN markers, especially their inconvenient storage and handling, consequent high costs, painful administration (local anesthesia should be avoided in the vicinity) as well as their long accumulation time requiring injection hours before surgery, radiation-free SLN markers could greatly improve hospital procedures (Bredell 2010; Süslü et al. 2022).

Beside that radioactive tracers are known to come along with the so-called “shine through” effect. SLNs located close to the primary tumor are not detected due to the strong radioactivity of the peritumoral region superimposing the much weaker signal from the adjacent SLN. It is considered the highest risk of failure in floor of mouth tumors, since lymph nodes in level I might not be detected (Bredell 2010; Kang et al. 2017; Park et al. 2018). To overcome this limitation in the head–neck region and to improve SLN visualization, additional Methylene blue-dye injection around the tumor has been performed. However, substantial disadvantages of blue-dye usage have been reported. Due to dark blue tissue colorization in the administered region, tissue differentiation is complicated and the visibility of resection margins is blurred (Pitman et al. 2002; Kim et al. 2021; Süslü et al. 2022). Thus, clinicians attempted to look for an alternative technique and introduced ICG-guided SLNB (Bredell 2010; Van Der Poel et al. 2011).

Compared to methylene blue, ICG is invisible to the human eye but fluorescent under NIR, produces low background signals, and is not absorbed by surrounded tissues (Süslü et al. 2022). Peng et al. (2015) report that the intraoperative detection rate was improved from 67 to 100% using ICG instead of Methylene blue dye. In the present study, only one patient suffered from a tumor located at the floor of the mouth; therefore, no information can be provided regarding the potential of ICG to diminish the “shine through effect”.

Due to its small molecular structure, ICG is quickly transported to the SLN, allowing immediate intraoperative injection just minutes before visualization (Polom et al. 2011). This can be considered a relevant advantage in these times where surgeries are frequently rescheduled, for example, due to short staffing. However, the migration of ICG to the lymph nodes may be delayed in patients suffering from impaired cervical capillary vessel structures following radiation or surgery (Van Der Vorst et al. 2013). This was not observed in our study. As all patients showed unscathed necks, the accumulation time varied from 1 to 3 min, and fluorescence time was long enough, so SLN mapping using ICG has been completed successfully in all cases.

Some authors have reported an ICG uptake in lymph nodes that were negative for ^99m^Tc, which was not the case in our patients (Van Den Berg et al. 2012b; Murase et al. 2015). This might be due to the rapid further distribution of ICG after accumulation in the sentinel lymph nodes, leading to an undesired spread of the marker. Given the accumulation time of 1–3 min found in our study, ICG injection should take place only after surgical preparation of the neck where the SLN is expected to be found.

Among the disadvantages of ICG are the impossibility of preoperative X-ray-based imaging for SLN localization, and the limited penetration of the fluorescence signal through subcutaneous fat and skin. Thus, the lack of a preoperative visualization of potentially affected lymph nodes and the inability to visualize deeper lying affected nodes may require larger incisions and surgical preparation to access the affected area (Bredell 2010; Murase et al. 2015). As the fluorescent signal can only be unproperly detected transcutaneous in the cervical region, the ICG–NIR procedure alone is considered unsuitable for SLNB in patients with oral cancer (Bredell 2010).

A combination of both SLN marker systems—either in the form of a hybrid marker or of sequential injections of radioactive and fluorescence-based markers—may provide relevant advantages in SLNB (Van Den Berg et al. 2012b; Digonnet et al. 2018). Visualization with an NIR camera improves the intraoperative SLN identification compared with a gamma probe alone, where surgeons must rely on acoustic feedback only (Kang et al. 2017). Another advantage of ICG bound to colloids compared to free ICG is the tracer retention. ICG alone has rapid kinetics and limited retention in SLN causing down-stream drainage to non-SLNs. As ICG bound to colloids is retained in the SLN to greater extend, it is reported to be favorable as it decreases the potential to miss the window for labeling during surgery (Christensen et al. 2016; Almhanedi et al. 2021).

The hybrid markers reduce the risk of lymph nodes being unnoted through to the shine-through effect, but with the administration the day before surgery, the fluorescence capacity decreases (Van Den Berg et al. 2012b; Süslü et al. 2022). The sequential injection reduces that risk, however, is still relying on a prior radioactive examination because of the lack of transcutaneous detection (Digonnet et al. 2018). The advantage of the sequential administration of ^99m^Tc- and ICG are considered in a better fluorescent signal compared with that of hybrid dyes without the expense of creating a new hybrid drug (Digonnet et al. 2018). Nevertheless, within both methods, the use of a fluorescence marker is reported to be of particular value when SLNs are located in close proximity to the primary tumor (Van Den Berg et al. 2012b; Digonnet et al. 2018). However, SLNB without radioisotope tracers, using CT lymphography in combination with ICG has been demonstrated to be as a feasible procedure, for neck management in cases of early tongue cancer (Ishiguro et al. 2020).

Given the fact that SLN identification does not depend on the level of experience of the injecting surgeon, both methods can be easily introduced into routine clinical practice. Furthermore, we want to underline that this study investigated concordance between radio-detected SLNs and subsequent optical SLN detection. This provides surgeons with reassurance to identify the right lymph nodes. In the future, further improvements in SLNB in OSCC can be expected by developing new markers. Especially markers that can be detected transcutaneous without the usage of a radioactive material will facilitate the broader usage of sentinel lymph-node biopsy in clinical practice.

The study showed a 100% agreement in sentinel lymph-node identification with ^99m^Tc and ICG regardless of the experience of the injecting surgeon and the exact peritumoral injection site. These findings highlight the robustness of the SLNB procedure.

## Data Availability

The data used to support the findings of this study are included within the article.
